# Capacity building in Nepal*

**Published:** 2016-12-05

**Authors:** Russell Dawe, Karl Stobbe, Yagya Raj Pokharel, Shrijana Shrestha

**Affiliations:** 1 Memorial University of Newfoundland; 2 McMaster University; 3 Patan Academy of Health Sciences

There’s an old saying: “Give a man a fish, feed him for a day; teach a man to fish, feed him for a lifetime.” But do we believe this? Many health care providers sacrifice their time and money to provide medical aid to underserved populations in remote environments. But is this helpful? Underserved populations need more than just another doctor. Short-term medical aid, with the exception of disaster relief situations, is often resource intensive for relatively little impact on the target population. Treating one patient at a time, though it matters to that one person, is like pouring water into a leaky bucket without patching the holes, since so many others remain untreated and underserved. Such an approach is unsustainable. Public health measures are crucial to sustainable disease-prevention and keeping the water out of the bucket in the first place. However, for those who do get sick we need to find a way to patch the holes and build capacity for health care.

Sustainability in health care is rising to the fore. Clinicians, educators, researchers, administrators and policy-makers are looking for ways to move beyond clinical care into capacity building. There’s a problem with the health care system’s capacity to meet the health needs of our domestic and international underserved populations, and capacity building is emerging as a core value in global health. That capacity building should indeed improve access to clinical care, but that does not necessarily mean the solution is for us to provide that care ourselves. Rather, we must address the question: How can we make a lasting contribution to the underserved populations we want to help? How can we help teach them to fish?

Physicians who travel to foreign places are often intrigued by the different health needs they encounter. However, a western doctor, just by his or her very presence, can unwittingly undermine local health care providers. If we invite patients to come to us instead of a local colleague, we imply that we offer something they lack and we may take valuable learning opportunities or needed income away from them. Furthermore, if we provide clinical care as part of a short-term trip, we may leave behind a gap in clinical service after we’ve finished. This runs the risk of creating a dependence upon our service, which we cannot sustain. If we really want to help we must find ways to make a more substantial and sustainable impact.

In Nepal, Patan Academy of Health Sciences (PAHS) is rising to the challenge: its strategy is rural medical education. Its five-year Bachelor of Medicine, Bachelor of Surgery (MBBS) program produces graduates equipped to provide basic primary care in rural Nepal. Graduates may provide health care as medical officers, or go on to complete a residency and become general practitioners (MDGPs; Nepal’s counterpart to Canada’s family doctors). Despite Nepal’s considerable population-health efforts to date (addressing micronutrient deficiencies, and immunizations, etc.), access to health care remains a problem. Such needs are not unique to Nepal: rural populations are under-resourced and underserved worldwide. The difference between western and developing countries here is a matter of magnitude. For example, Nepal has approximately 0.2 physicians per 1,000 population, whereas Canada has 2.1.^1^ Health care provided in a primary care (as opposed to specialist care) model is associated with more equitable distribution of health, improved health outcomes, and lower total health care costs.^2^ In many countries, Nepal included, patients with health issues that fall under the scope of a general practitioner often receive specialist care instead, despite the increased cost associated. PAHS, therefore, is training general practitioners as its strategy to improve access to cost-effective care. When we invest in education, we have the opportunity to increase the number and quality of physicians that graduate, as well as influence the likelihood that they will practice primary care in an area of need. That’s just patching holes in the bucket.

In addition to extensive rural exposure, PAHS’s innovative curriculum emphasizes upstream population health issues that are fundamental to capacity building. MBBS students receive in-depth community health exposure interwoven throughout their medical curriculum. First year students spend one week in a slum community and one week as a residential posting, living with a village household, to learn the social determinants of health through firsthand experience. Their community health exposure continues to increase throughout the program. In their fifth year, MBBS students spend twenty weeks in a rural or peripheral hospital and public health unit with a small group of classmates and one MDGP. In this rural service-learning rotation, PAHS’s senior students develop the clinical competence and confidence to practice in a low-resource environment. Through their program’s rural focus, more family doctors can be expected to practice in rural Nepal. That’s capacity building.

What can we do as Canadian or western health care providers? In 2013, Dr. Karl Stobbe (McMaster University) began recruiting rural Canadian family doctors to help in Nepal. Dr. Stobbe and his team asked PAHS what would be helpful and were advised to help teach the medical students. These Canadian family doctors, supported by Academics Without Borders (AWB) and the Society of Rural Physicians of Canada (SRPC), began a partnership with PAHS, based on a common mission to build capacity for primary care among underserved populations.

During the MBBS’s twenty-week rural rotation, these Canadian family doctors go to rural Nepal to help teach those senior medical students, while modelling a passion for rural medicine. With a full clinical load, the local Nepali physician on-site has limited time to spend with her or his learners. However, the visiting Canadian physicians don’t take on a clinical load. Rather, they provide clinical bedside teaching, observing the student-patient interaction, and giving feedback on basic clinical skills. In this way, visiting physicians contribute to the students’ learning while providing academic relief for a short time to the local Nepali faculty. The visiting physicians do not directly treat patients at all.

The beauty of using clinical bedside teaching as a tool for capacity building is that it plays to our strengths as family doctors. You don’t have to be an expert in parasitology in order to help a learner take a thorough history. You don’t have to be competent in managing multi-drug resistant tuberculosis in order to help students broaden their differential diagnosis. In Canada, clinical skills represent every family doctor’s bread and butter and are foundational to our medical training. And that’s exactly what their learners need. The Nepali students have already seen plenty of communicable disease so we don’t have to become experts in a new field before we have something to offer. They know how to manage tropical medicine in an under-resourced practice. What they need are teachers with time to help them hone their fundamental clinical skills, not their knowledge of local medical conditions.

Additionally, rural family doctors set an example for PAHS’s students, not just in clinical skills, but also in their commitment to rural practice. In Nepal, medical graduates are often reluctant to practice in a rural or remote environment due to a lack of resources at these sites and/or a lack of confidence in their own ability to practice independently in such an environment. So when rural Canadian physicians show up and demonstrate that we feel rural family practice is a calling and a career, it teaches something that cannot be learned from a textbook: the value and beauty of rural medicine.

In the process of this collaborative teaching and role modelling, all parties benefit. Visiting physicians still learn from clinical exposure, even though they’re not providing direct patient care. Local faculty and students appreciate the vote of confidence that visiting physicians provide when we encourage them in their work. Finally, by providing clinical bedside teaching, visiting physicians are not replacing the local caregivers but instead affirming and strengthening them.

In summary, clinical bedside teaching that models a commitment to rural practice provides an opportunity to build capacity while both parties learn from the experience. “Teach a man to fish, feed him for a lifetime.” Clinical bedside teaching on its own is not going to fix the leaky bucket of our world’s health care systems, but it’s addressing the right question. If we are going to make a sustainable difference, we must ask the right questions and collaborate across traditional boundaries of geography and politics. The communities and programs we partner with, like PAHS, are experts to be consulted and supported, not incompetents to be replaced. Partnerships intentional about patching health care’s leaky bucket may provide the greatest hope to our underserved populations.
